# LC-HRMS-Based Identification of Transformation Products of the Drug Salinomycin Generated by Electrochemistry and Liver Microsome

**DOI:** 10.3390/antibiotics11020155

**Published:** 2022-01-25

**Authors:** Lisa Knoche, Jan Lisec, Tanja Schwerdtle, Matthias Koch

**Affiliations:** 1 Department of Analytical Chemistry and Reference Materials, Bundesanstalt für Materialforschung und -prüfung (BAM), Richard-Willstätter-Straße 11, 12489 Berlin, Germany; lisa.knoche@bam.de (L.K.); jan.lisec@bam.de (J.L.); 2 Institute of Nutritional Science, University of Potsdam, Arthur-Scheunert-Allee 114-116, 14558 Nuthetal, Germany; tanja.schwerdtle@uni-potsdam.de; 3 German Federal Institute for Risk Assessment (BfR), Max-Dohrn-Str. 8-10, 10589 Berlin, Germany

**Keywords:** salinomycin, ionophore antibiotics, transformation product, electrochemistry, rat/human liver microsomes, HRMS

## Abstract

The drug salinomycin (SAL) is a polyether antibiotic and used in veterinary medicine as coccidiostat and growth promoter. Recently, SAL was suggested as a potential anticancer drug. However, transformation products (TPs) resulting from metabolic and environmental degradation of SAL are incompletely known and structural information is missing. In this study, we therefore systematically investigated the formation and identification of SAL derived TPs using electrochemistry (EC) in an electrochemical reactor and rat and human liver microsome incubation (RLM and HLM) as TP generating methods. Liquid chromatography (LC) coupled to high-resolution mass spectrometry (HRMS) was applied to determine accurate masses in a suspected target analysis to identify TPs and to deduce occurring modification reactions of derived TPs. A total of 14 new, structurally different TPs were found (two EC-TPs, five RLM-TPs, and 11 HLM-TPs). The main modification reactions are decarbonylation for EC-TPs and oxidation (hydroxylation) for RLM/HLM-TPs. Of particular interest are potassium-based TPs identified after liver microsome incubation because these might have been overlooked or declared as oxidated sodium adducts in previous, non-HRMS-based studies due to the small mass difference between K and O + Na of 21 mDa. The MS fragmentation pattern of TPs was used to predict the position of identified modifications in the SAL molecule. The obtained knowledge regarding transformation reactions and novel TPs of SAL will contribute to elucidate SAL-metabolites with regards to structural prediction.

## 1. Introduction

Salinomycin (SAL) is an ionophore antibiotic that shows antibacterial, antifungal, antiparasitic, and antiviral properties [1,2]. SAL is commercially used as a veterinary drug to treat and prevent coccidiosis in poultry farming. Furthermore, SAL shows potential as growth promoter in modern animal husbandry (usage not allowed in the EU). In 2009, Gupta et al. observed that SAL selectively negatively impacts on breast cancer stem cells [3], inducing intensive studies of SAL as a novel therapeutic agent in different human cancer types (e.g., breast, colon, and leukemia) [4].

**Figure 1:**
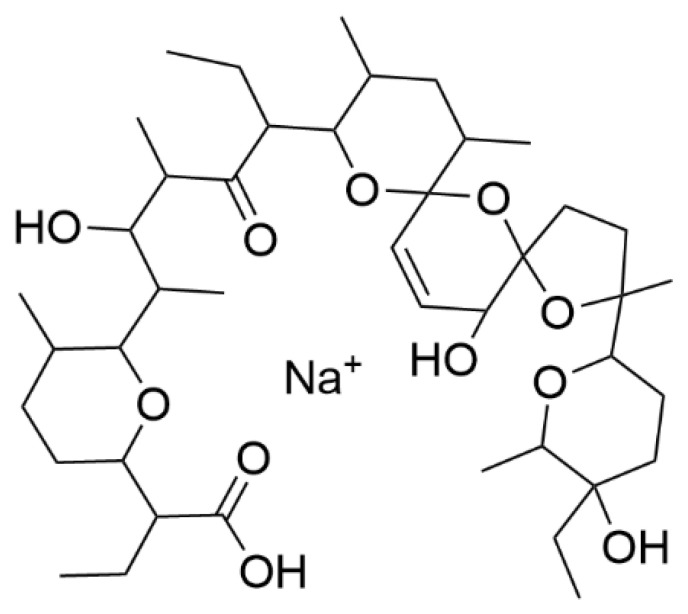
Chemical structure of salinomycin (SAL) as sodium-complex.

The chemical structure of SAL consists of a polyether skeleton with five polyether rings. Three rings form a unique tricyclic spiroketal system, whereby the middle ether ring has a double bond. Similar to other ionophore antibiotics, SAL has a carboxylic group and an ether-ring with a hydroxy-group in terminal positions [1,4]. These specific structural properties lead to the occurrence of a pseudo cyclic SAL complex with metal cations (see Figure 1). The selectivity of forming complexes is dependent on the polar metal cation. SAL shows the highest affinity for complexation with potassium (K^+^) followed by sodium (Na^+^). The formed SAL-complex is lipophilic and can enter lipid bilayers to transport cations across them [1,4].

A generic term for metabolic and environmental degradation products is transformation products (TPs) [5]. All TPs of SAL are possible residues in foodstuff or in environmental matrices. They occur at low levels (up to trace levels), and liquid-chromatography high-resolution mass spectrometry (LC-HRMS) enables the identification of novel TPs using non-target approaches [5,6]. TPs are expected to exhibit different properties compared to their precursors, which might include higher toxicity and persistence. Different laboratory approaches are used to simulate (generation of TPs under laboratory conditions is termed simulation) and identify TPs [7].

Public data about SAL derived TPs are partly available. A comprehensive overview of different experimental investigations with regard to identification of metabolites/degradation products of SAL is given in Table 1. The results are ordered by their transformation reaction. Metabolites of SAL are mostly generated by (+O) (hydroxylation; +OH; as mono-, di-, and tri hydroxylation). This is in accordance with the scientific opinion over SAL-sodium of the European Food and Safety Authority, where it is mentioned that SAL is extensively metabolized by chicken, with mono- and multi-hydroxylated or keto-derivates of SAL [8]. In recent years, the group of Olejnik investigated metabolites of SAL intensively. They identified metabolites in tests with human hepatoma cells (HepG2) (14 metabolites) [9], primary human hepatocytes (PHH) (20 metabolites) [10], and rat primary hepatocytes (PRH) (16 metabolites) [11] and rat hepatoma cells (FaO) (three metabolites) [11]. In contrast, based on the widely usage of SAL as veterinary drug, the degradation behavior including degradation products is well-investigated. A degradation product with *m/z* of 531.3 is identified by several studies, and the degradation process is based on C-C cleavage (mainly at the β-hydroxy-ketone position) [12].

In general, transformation processes are based on chemical mechanisms such as redox or radical-based reactions. The use of electrochemistry (EC) is well-established to simulate redox-reactions and generate TPs [20,21]. A widely used instrumental set-up is the combination of an electrochemical reactor (ECR) with mass spectrometry (MS). Inside the cell of the ECR, an applied electrical potential induces different transformation reactions. The ECR-MS can be used in two modes, online or offline. For the online mode, a direct coupling between the ECR and the MS enables the detection of stable and transient TPs [22]. For offline measurements, the eluent is collected after the ECR and is analyzed in a second step. The absence of complex matrices usually simplifies TP identification. Depending on the selected potential (positive or negative) in the EC cell, oxidation or reduction reactions can be induced, allowing the simulation of a broad reaction spectrum [20]. The simulation of cytochrome P450-mediated metabolic reactions of drug metabolism by electrochemistry has been published by several studies [22,23].

In contrast to EC-based approaches, metabolism tests of different complexity are known to simulate metabolic reactions in the laboratory ranging from in vivo/in vitro experiments (e.g., transgenic cell lines, primary hepatocytes, and liver slices) to cell free incubation with human (HLM) or rat liver microsomes (RLM) [24]. After incubation, the metabolites are isolated and analyzed by LC-HRMS, which allows an accurate mass determination of the TPs resulting in molecular formulas. The modification reactions can often be derived from the molecular formulas [6]. Additionally, MS/MS fragmentation patterns are investigated to obtain structural information about the TPs.

The aim of this study was to investigate the transformation behavior (modification reactions) of SAL by the generation of TPs using electrochemistry and liver microsome incubation and to identify the resulting TPs. To this end, HRMS was used to improve identification in comparison to the low-resolution MS data generated in other studies. The focus was placed on the suspected-target analysis of the HRMS data of measured TPs to obtain as much information as possible (in relation to sum formula, modification reaction, and structure). The newly found EC and HLM/RLM-TPs are compared with each other and degradation products of SAL described in the literature.

## 2. Results and Discussion

### 2.1. Electrochemical Investigation

The oxidation behavior of SAL and identification of SAL-TPs was studied by two different settings: First, the online-mode consisting of an ECR coupled to an ESI-source of a HRMS, and, secondly, an offline set-up combining ECR and LC-HRMS to obtain further information regarding structural proposals of the generated TPs.

At the beginning, the experimental conditions for the electrochemical generation of SAL-TPs were optimized with respect to several parameters (solvent, modifier, and working electrode). More detailed information is given in ESM (Supplementary Materials Table S1). The best reproducibility and highest number of TPs were achieved by using a GC working electrode and a solvent mixture of methanol:acetonitrile:water (3:1:1, *v*/*v*/*v*) with 1 mM ammonium formiate. The SAL-containing solution was transferred to the electrochemical cell where an electric potential was applied (0 to 2.5 V).

**Figure 2:**
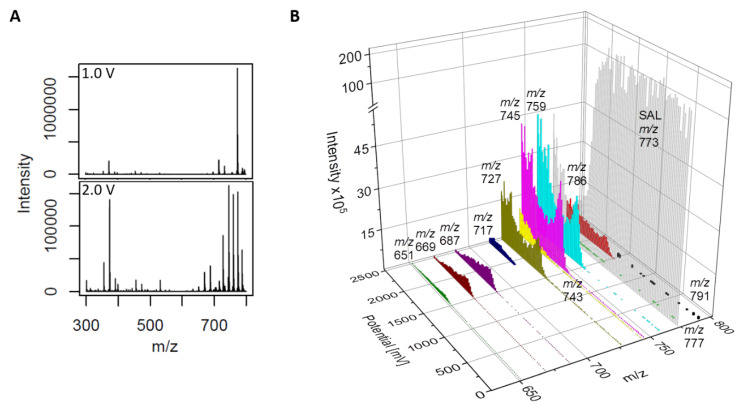
Online ECR/HRMS measurements of SAL (**A**) Mass spectra of SAL at a potential of 1.0 V (upper spectrum) and 2.0 V (lower spectrum). (**B**) 3D mass voltammogram of SAL. Mass traces (*m/z*) of the TPs of SAL as [M + Na]^+^ in dependence of the applied oxidation potential ramped from 0 to 2.5 V.

The results are displayed in Figure 2A, consisting of two mass spectra at different potential-ranges (around 1.0 and 2.0 V). At the higher potential (lower spectrum), several additional *m/z* peaks could be obtained. A more detailed visualization is given by the three-dimensional mass voltammogram (Figure 2B), presenting the mass spectra (*m/z* 630 to 800) against the applied potential (0.0 to 2.5 V). The potential-dependent intensity course is visualized for SAL (*m/z* = 773, decreasing with increasing potential) and 11 increasing *m/z*-traces. The *m/z*-traces may represent potential EC-TPs and showed different starting occurrence and intensity courses in relation to maximum intensity. The term EC-TPs is used for all eleven *m/z*-traces independent of whether the *m/z*-trace is generated by EC. Exact masses as well as the corresponding modifications of all products are listed in Table 1. Sum formulas were calculated based on the measured accurate mass. This allows drawing conclusions about the type of causal SAL-modification reactions. Different modification reactions were found; the most prominent was the decarbonylation (−CO) for 10 TPs. Further modification reactions were decarboxylation (−CO_2_), dealkylation (−C_x_H_y_), (de-)hydrogenation (+/−H), dehydration (−H_2_O), oxidation (+O), and reduction (−O). Additionally, SAL-TPs were found by forming complexes with a second cation (sodium (+Na) and/or ammonium (+NH_4_^+^)). One intense TP is EC-TP-5 with *m/z* 745 showing decarbonylation, the most prominent modification. Additionally, a high intensity shows EC-TP-4 with *m/z* 759 (reduction and hydrogenation). The formation of these TPs started at a potential around 1.5 V. The EC-TP-2 with *m/z* 786 showed an adduct formation next to the decarbonylation with ammonium and a second sodium ion. In addition to adduct formation of EC-TP-4, the dehydrated EC-TP-7 with *m/z* of 727 was existing. Around the same potential (1.5 V), EC-TP-9, 10, and 11 were also occurring. These three TPs showed a similar pattern of intensity and ion abundance decreasing with *m/z*. The mass difference of 18 Da between TP9/TP10 and TP10/TP11 indicates a loss of water. Further TPs with *m/z* of 717 (EC-TP-8), 743 (EC-TP-6), 777 (EC-TP-3), and 791 (EC-TP-1) appeared at a potential around 2.0 V. The EC-TP-1 was the ammonium adduct of the EC-TP-3 (decarbonylation and di-oxidation). Dehydrogenation of EC-TP-5 leads to EC-TP-6 and a dealkylation to EC-TP-8.

While the online-mode allows an easy identification of interesting *m/z*-traces, it does not allow distinguishing whether these traces represent TPs generated inside the ECR or by-products due to in-source-fragmentation during ionization [25]. To obtain information about the generation and stability of occurring *m/z*-traces, additional offline LC-HRMS measurements of the EC-treated SAL-solution were performed. MS/MS fragmentation was used to obtain further structural information about the generated TPs. In comparison to the SAL-standard chromatogram, the chromatogram of the EC-treated SAL-solution showed two additional peaks. The online measurements result in 11 *m/z*-traces (Table 2), and only two of them, EC-TP-5 (*m/z* 745.486) and EC-TP-7 (*m/z* 727.476), were found also in offline LC-HRMS measurements. Upon closer inspection, we found that four of the *m/z*-traces were co-eluting at low intensities together with EC-TP-5 (EC-TP-10), EC-TP-7 (EC-TP-6 and EC-TP-11), or SAL (EC-TP-1), respectively (see Figure S1). This indicates that these *m/z*-traces are potential in-source-fragments.

**Figure 3:**
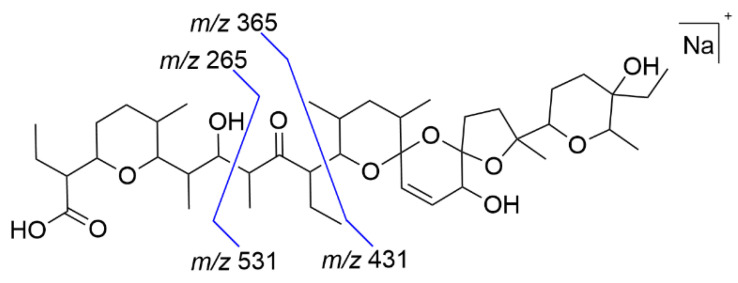
Simplified presentation of the fragmentation pattern of SAL (proposed by Miao et al. [26]). Four major fragments were used for structural prediction.

**Figure 4:**
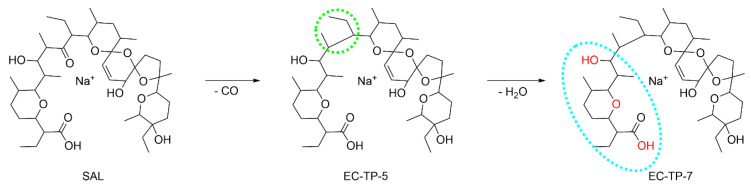
Proposed structures of the TPs derived from EC experiments with SAL using GC electrode. The dotted circles mark the areas of modification. The red highlighted oxygen-atoms of EC-TP2 could be involved in a loss of water.

The two stable TPs of SAL were measured by LC-HRMS, and the MS/MS fragmentation of EC-TP-5 and EC-TP-7 is used to localize the region of SAL, where the EC induced modification is occurring. The evaluated MS/MS data were compared to previously described ESI-fragmentation of SAL by Miao et al. [26] A shortened fragmentation scheme is presented in Figure 3, and the detailed fragmentation scheme is given in ESM (Supplementary Materials Figure S2 and Table S2). The main fragmentation of SAL is occurring at both sides of the carbonyl function by a β-cleavage of the C-C bond. The resulting fragment pairs of *m/z* 531 + *m/z* 265 and *m/z* 431 + *m/z* 365 serve as basis for the evaluation. The associated MS/MS fragments to the fragmentation pathway are given in Table S3 (see Supplementary Materials). The EC-TP-5 has an accurate mass of *m/z* 745.486 that corresponds to a decarbonylation of SAL. The evaluation of the MS/MS data identified the modification at fragment *m/z* 365, but the fragment of *m/z* 265 is available unchanged. It is assumed that the carbonyl moiety of SAL is eliminated, visualized in Figure 4. The EC-TP-7 (accurate mas *m/z* 727.474) is modified by decarbonylation and dehydration. It seems that the decarbonylation is occurring at the same position as EC-TP-5. The placement of the dehydration is expected to fragment *m/z* 265, and three positions (hydroxy-groups and ether-ring) are possible (see Figure 4).

The obtained results showed eleven *m/z*-traces of SAL, whereby six were found in the offline measurements distributed at two peaks. The decarbonylation was the main-modification reaction occurring by EC for SAL and the EC-TP-5 showed exactly these. Based on the data the region of the modification was identified and a structure predicted. The decarbonylation reaction as a modification of SAL was previously undescribed. The comparison between the online found *m/z*-traces and offline found TPs resulted in five missing EC-TPs. Except for EC-TP-4 (*m/z* 759), all other missing *m/z*-traces show only a low intensity. Potential reasons for the absence are (i) low stability of the TPs and loss of adduct formation during storage, (ii) stability of adduct-formation is depended on matrix conditions (in online-measurements the ammonia concentration is much higher than in offline-mode), and (iii) differences in the amount of in-source fragmentation (online measurements might enhance fragmentation compared to offline-mode).

### 2.2. Liver Microsomes Assay

Liver microsomes assay were performed with HLM and RLM. SAL is incubated either with HLM or RLM in potassium phosphate buffer; afterwards, the samples are analyzed by LC-HRMS. In Table 3, all obtained TPs are listed including the accurate mass and suggested modification. All TPs were more polar than SAL, indicated by the shorter retention time under reversed-phase LC conditions. In total, five TPs for the RLM incubation and 11 TPs for the HLM incubation were detected. Four TPs were identical, having the same accurate mass and retention time (R1-H4, R2-H6, R3-H9, and R4-H11). In general, the conversion of SAL was higher for the HLM-incubation than for the RLM-incubation. The main modification reaction was oxidation (+O) occurring as mono-, di-, and tri-oxidation. The oxidation can occur as hydroxylation (+OH/−H) or as epoxidation (+O). Epoxidation is possible at the double bound of the spiro-ketal ring system. Attention should be paid to the potassium TPs in which sodium is replaced by potassium. In the literature, SAL complexes with different mono- and divalent cations are described, with potassium exhibiting the highest affinity to SAL [4]. The evaluation of the accurate mass was more complex due to the sodium–potassium exchange. The mass difference between sodium (*m/z* 22.9898) and potassium (*m/z* 38.9637) is 15.9739, causing confusion with oxygen (*m/z* 15.9949) (dm = 0.021). If HRMS is not used, there will be a risk of determining a hydroxylation instead of an Na–K exchange. An example was TP-H2 (*m/z* 819.4296) and TP-H8 (*m/z* 819.4507); TP-H2 as the potassium-complex showed a di-hydroxylation and TP-H8 as sodium-complex a tri-hydroxylation, both in combination with di-dehydrogenation.

**Figure 5:**
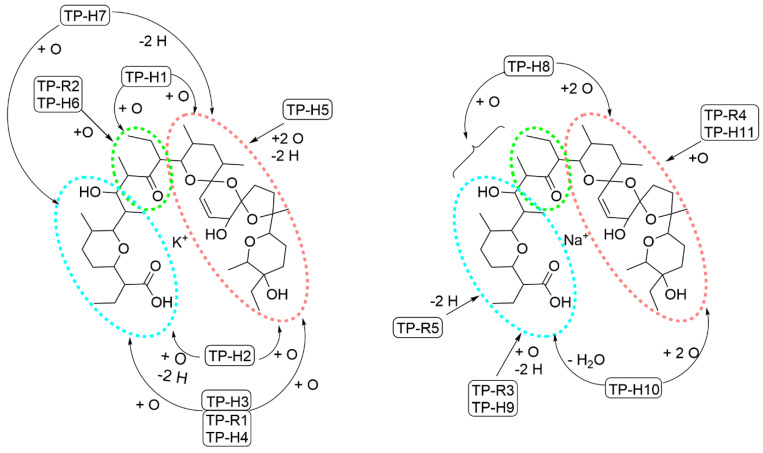
Overview of all TPs of SAL found in/by RLM and HLM assays; left hand; the potassium-based TPs; right hand; the sodium-based TPs. Numbers correspond to compounds presented in Table 3. Three regions of SAL are highlighted as modification area by dotted circles, the blue circle contains the carboxy-end, the red circle contains the spiroketal-end, and the green circle the carbonyl group.

The HRMS results also included data from MS/MS-analyses. The aim of the evaluation was to identify structural parts where the modification is located. For the RLM/HLM TPs, it was not possible to determine the exact position in the SAL molecule where the modification reaction takes place. The smallest obtained fragments were still too large to draw definite conclusions about structural modifications. In particular, even when modifications such as hydroxylation could be mapped to the same region of SAL for different TPs (Figure 5), it was not possible to confirm whether or not the modification took place at the same C-atom. For example, TP-H3 and TP-H7 showed both a hydroxylation at the carboxy-group (blue circled region), but the MS/MS-fragments did not allow specifying a common C-atom. In principle, the eligible fragments are based on the main MS-fragments of SAL (see Figure 3).

An overview of all HLM/RLM-TPs is displayed in Figure 5, where SAL is divided into three structural regions (carboxy-, spiroketal-, and carbonyl- groups). The main observed modification was oxidation (mostly hydroxylation) and, in the end, several positions of the fragments were possible for hydroxylation. In general, the C-atom for hydroxylation was more dependent on intrinsic reactivity (ter. C > sec. C > prim. C) than steric constraints. [27] Analogues to the EC-TPs the MS/MS-data with associated fragment part of the RLM/HLM-TPs are given in Supplementary Materials Tables S4–S6.

Ten structural different TPs were identified, five forming a potassium-complex and five forming a sodium-complex. Three TPs showed an accurate mass of 821.445 (K-complex, di-oxidation) eluting at different retention times. TP-H1 showed a different fragmentation-pattern than TP-H3 and TP-H4/R1. It is assumed that TP-H3 and TPH4/R1 are stereoisomers of each other because of their similar fragmentation pattern and their small retention time difference. TP-H2 and TP-H5 (+O/−H) showed an accurate mass of 819.429, and the fragmentation pattern results in different positions for the modification reactions. TP-H8 (*m/z* 819.451) was sodium-based, and a tri-oxidation was occurring. The intensity is very low, resulting in less MS/MS-fragmentation and less structural information. The calculated molecular formula of TP-H10 (*m/z* 787.461) indicates oxidation and di-dehydration as modification reactions, but the evaluation of the fragmentation pattern gave evidence for a di-oxidation with dehydration at another region of SAL. Some K-TPs and Na-TPs showed the same modification (e.g., TP-H6 and TP-H11/R4; TP-H5, TP-H7, and TP-H9/R3), but a comparison showed that the modification reactions are occurring at different structural parts of SAL. For example, the oxidation takes place at the carbonyl-fragment for TP-H6 (K-based) and for TP-H11/R4 (Na-based) at the spiroketal-fragment. Of course, there were modifications at the same fragment for both types as oxidation at the spiroketal (e.g., TP-H1 to H5 and TP H8, H10, and H11).

The identification of metabolic degradation products of SAL is sparsely discussed in literature so far. The group of Olejnik et al. conducted studies with human and rat cells and identified up to 20 metabolites of SAL by LC-MS/MS. Hydroxylation as mono-, di-, and tri-hydroxylation was identified as main modification reaction. This is in accordance with the results of the RLM/HLM-TPs described above. A further comparison is impossible, based on different analytical measurement techniques (HRMS and TripleQuad MS). The mass accuracy of a Triple Quad-system is unsatisfactory to distinguish the mass difference between a Na–K exchange and hydroxylation (0.02 Da).

## 3. Materials and Methods

### 3.1. Chemicals

Salinomycin monosodium salt hydrate (purity 93%, VetranalTM) was purchased from Sigma-Aldrich (Steinheim, Germany). KH_2_PO_4_ was purchased from Chemsolute (Renningen, Germany) and K_2_HPO_4_ from Carl Roth (Karlsruhe, Germany). NADPH tetrasodium salt was obtained from Carl Roth (Karlsruhe, Germany). Potassium biphthalate was obtained by Fluka Chemika (Buchs, Switzerland). Acetonitrile and methanol were purchased from Chemsolute (Renningen, Germany). Ammonium formate was purchased from Fluka Chemika (Buchs, Switzerland) and formic acid from Merck (Darmstadt, Germany). Ultrapure water was produced by a Purelab Flex 2 system, ELGA Veolia Water technologies (Celle, Germany). All standard chemicals were of p.a. grade an all solvents (acetonitrile, methanol) of LC-MS grade.

### 3.2. ECR/ESI-HRMS

The electrochemical system consisting of a ROXYTM potentiostat (Antec Scientific, Zoeterwoude, The Netherlands) and an electrochemical flow-through cell. The instrument was controlled via Dialogue Elite software (Antec Leyden) version 2.0.0.81. Online coupling of the electrochemical cell to an electrospray ionization source of a TripleTOF^®^ 6600 Quadrupole Time-Of-Flight (QTOF) mass analyzer (Sciex, Darmstadt, Germany) was used for recording of mass voltammograms. The QTOF system was controlled via Analyst^®^ TF1.8.0 including data processing. Additional in-house scripts of the statistical working environment R (REF) [28] were used for data processing. Graphical representation of the three-dimensional mass voltammograms were created by Origin 2019 (OriginLab, Northampton, MA, USA). The SAL-solvent mixture consisting of 20 µM SAL in methanol:acetonitrile:water (3:1:1; *v*/*v*/*v*) with 1 mM ammonium formiate, was passed through the cell by a Legato^®^ 110 dual rate system syringe pump (KD Scientific, Hollison, MA, USA) with a flow rate of 40 µL/min. The EC cell consist of a three-electrode arrangement including a titanium auxiliary electrode (inlet-block of the cell), a HyREFTM-reference electrode (Pd/H2), and a glassy carbon (GC) working electrode. The applied potential was ramped between 0 and 2.5 V with a scan rate of 10 mV/s controlled by the potentiostat. The GC-working electrode was activated before each measurement by a manufacturer-provided pulse-cleaning program. The EC-cell was connected to the ESI-HRMS, and the experimental parameters for MS-detection are given in Table 4. A mass voltammogram was recorded three times to ensure the reproducibility of the measurements. Control measurements were performed by using the solvent without analyte. Next to the online measurements, aliquots were collected in a HPLC-vial from the EC-cell and were used for further offline LC-HRMS measurements.

### 3.3. Microsomal Assay

Rat liver microsomes (RLM) were purchased from Thermo Fisher Scientific (Pittsburgh, PA, USA). The RLM were prepared from Sprague Dawley male rats with a protein concentration of 20 mg/mL and a CYP450 content of 319 pmol/mg protein. The total protein content and CYP450 concentrations were provided by the manufacturer. Human liver microsomes (HLM) were purchased from Thermo Fisher Scientific (Pittsburgh, PA, USA). The HLM were prepared from 20 human female donors with a concentration of 20 mg/mL and a P450-specific content of 309 nmol/mg protein (concentration provided by the manufacturer). Incubations with liver microsomes (LM) were carried out in a volume of 200 µL. Microsomes (1.0 mg/mL microsomal protein) were mixes with 0.1 M potassium phosphate buffer (pH 7.4), and 0.01 mM MgCl_2_. First, a pre-incubation for 5 min at 37 °C took place; then, SAL (6.25 µmol/L) dissolved in ACN (total ACN amount < 3%) and 0.6 mM NADPH was added to the mixture to start the enzymatic reaction (incubation time: 90 min, 37 °C, 800 rpm). To stop the reaction, 50 µL ACN (−20 °C) was added and the sample was mixed thoroughly for 30 s (final SAL concentration: 5 µM). Afterwards, the incubation mixture was centrifuged at 12 rpm. The supernatant was analyzed by LC-HRMS (Agilent Technologies, Waldbronn, Germany/Sciex, Darmstadt, Germany). Control incubations, where the amount of NADPH was replaced through potassium buffer, were performed in duplicate. The reaction was performed in triplicate.

### 3.4. LC-HRMS

The LC-HRMS measurements of the electrochemical and microsomal tests were performed by LC-HRMS using a TripleTOF^®^ 6600 Quadrupole Time-Of-Flight (QTOF) mass analyzer (Sciex, Darmstadt, Germany) connected to an Agilent 1290 Infinity II (Agilent Technologies, Waldbronn, Germany), consisting of a 1290 Infinity II multisampler, a 1290 Infinity II flexible pump, a 1260 Infinity II diode array detector HS, and 1290 Infinity II multicolumn thermostat. The installed software for operation of the system is Analyst^®^ TF1.8.0 (AB Sciex), and the data were processed by SciexOS and using in-house scripts of the statistical working environment R (REF) [28]. The analytical column was a ZorbaxEclipse Plus C18, particle size 1.8 µm, 50 mm × 2.1 mm (Agilent Technologies, Waldbronn, Germany), and the column oven was set to 25 °C. A mobile phase of (A) H_2_O + 0.1% formic acid and (B) ACN + 0.1% formic acid was used for separation of the different samples. The injection volume was 2 µL. The flow rate of the mobile phase was 0.8 mL/min, and a gradient program was used for the separation. Starting was 50% of B, and, within 0.5 min, it was raised to 90%. After 3.5 min, it was decreased to 50%, and the column was re-calibrated for 3 min. The conditions for the TTOF are listed in Table 5, an information-dependent acquisition (IDA) was included for MS/MS experiments. The mass accuracy of the used Sciex TTOF is <2 ppm and was confirmed via a tuning run before any measurement. To assign a potential sum formula and chemical structure to a measured ion mass, all possible sum formulas within 3 ppm deviation around the respective *m/z* were calculated, allowing only the elements C, H, O, Na, and K together with N, to account for adduct formation in the ESI ion source. If more than one structural proposal remained, a fit of the isotopomer distribution (sigma value) was used to rank candidates. Additionally, the modifications in relation to SAL were calculated divided into gain and loss of atoms. Then, the best proposal was selected with focus on reasonable gain and loss groups. MS/MS spectra, which were acquired in IDA mode, allowed a further inference of structural confirmation of the precursor molecule.

## 4. Conclusions

In summary, the application of electrochemistry and assays with rat and human liver microsomes were used to generate TPs of the drug SAL. The online ECR/HRMS set-up led to 11 EC-TPs occurring by applied potential. Two of them were stable and could also be found by offline LC-HRMS measurements. The liver microsome assay resulted in five RLM-TPs and eleven HLM-TPs. The result of four identical TPs leads to the conclusion that RLM is dispensable because of the nearly completely redundant TPs with HLM. The evaluation of the accurate masses of the TPs shows decarbonylation as main modification reaction type for electrochemistry and oxidation (hydroxylation or epoxidation) as the main modification reaction for liver microsome assays. While additional MS/MS data led to predicted structures for both EC-TPs, the observed modifications of liver microsome TPs could only be assigned to a specific region of SAL. This is the first study that used accurate mass determinations to characterize TPs of SAL leading to the identification of 14 structurally different TPs. Noteworthy are the cation-exchanged TPs of SAL. The TPs found by online ECR/HRMS showed ammonia- and sodiated-sodium-complexes. However, the potassium-based TPs of the liver microsome assay(s) are more prominent. The results of our study contribute to a better understanding of the biotransformation process of SAL and to improve/facilitate the residue analysis of SAL in biological and environmental samples.

## Figures and Tables

**Table 1:** Overview of literature described metabolites or degradation products of salinomycin ordered by the transformation reaction.

Transformation Reaction	Experiment	References
*m/z* 531 (C-C cleavage, β-hydroxy-ketone position)	degradation by poultry litter	[13]
	degradation by broiler litter	[14]
	manure storage	[15]
	hydrolysis (acid-catalyzed)	[16]
	treatment with formic acid	[17]
	photodegradation	[18]
	transformation in soil	[19]
	microbial decomposition	[12,14]
*m/z* 265 (C-C cleavage)	hydrolysis (acid-catalyzed)	[16]
	treatment with formic acid	[17]
	photodegradation	[18]
Hydroxylation (+O)	human hepatoma cells (HepG2)	[9]
	primary human hepatocytes (PHH)	[10]
	rat primary hepatocytes (PRH)	[11]
	rat hepatoma cells (FaO)	[11]
Hydroxylation + Demethylation	photodegradation	[18]
	human hepatoma cells (HepG2)	[9]
	primary human hepatocytes (PHH)	[10]
Di-/Tri-hydroxylation	photodegradation	[18]
	primary human hepatocytes (PHH)	[10]
	rat primary hepatocytes (PRH)	[11]
Dehydrogenation	rat primary hepatocytes (PRH)	[11]
Isomeric changes	hydrolysis (acid-catalyzed)	[16]

**Table 2:** The intensity is calculated in relation to the highest TP-signal (*m/z*-trace 759) vs. (very strong) > 60%, s (strong): 40–60%, m (moderate): 20–40%, w (weak) 10–20%, vw (very weak) < 10%).

EC-TP	Mass Meas.	Mass Calc.	Sum Formula	Suggested Modification	Intensity
1 *	791.4801	791.4795	C_41_H_70_O_12_NNa	−CO, +2O, −4H, +NH_4_	vw
2	786.5116	786.5108	C_41_H_74_O_10_NNa_2_	−CO, +NH_4_, + Na	m
3	777.4804	777.4764	C_41_H_70_O_12_Na	−CO, +2O	vw
4	759.4956	759.5000	C_42_H_72_O_10_Na	−O, +2H	vs
5 ^§^	745.4859	745.4866	C_41_H_70_O_10_Na	−CO	vs
6 *	743.4710	743.4710	C_41_H_68_O_10_Na	−CO, −2H	m
7 ^§^	727.4764	727.4761	C_41_H_68_O_9_Na	−CO, −H_2_O	s
8	717.4552	717.4553	C_39_H_66_O_10_Na	−CO, −C_2_H_4_	vw
9	687.4817	687.4811	C_39_H_68_O_8_Na	−CO, −CO_2_, −CH_2_	w
10 *	669.4732	669.4706	C_39_H_66_O_7_Na	−CO, −CO_2_, −CH_2_, −H_2_O	w
11 *	651.4621	651.4600	C_39_H_64_O_6_Na	−CO, −CO_2_, −CH_2_, −2H_2_O	vw
SAL	773.4816	773.4815	C_42_H_70_O_11_Na		

* EC-TPs indicated by an asterisk are potential ESI in source fragments. ^§^ These EC-TPs have been confirmed in offline measurements.

**Table 3:** Detected TPs of SAL in a liver microsome assay. The intensity is calculated in relation to the highest TP-signal vs (very strong) > 60%, s (strong): 40–60%, m (moderate): 20–40%, w (weak) 10–20%, vw (very weak) < 10%).

	rt [s]	Mass Meas.	Mass Calc.	Sum Formula	Suggested Modification	Intensity
HLM						
TP-R1	65.20	821.4456	821.4453	C_42_H_70_O_13_K	−Na, +2O, +K	vw
TP-R2	81.35	805.4510	805.4504	C_42_H_70_O_12_K	−Na, +O, +K	m
TP-R3	91.57	787.4582	787.4608	C_42_H_68_O_12_Na	−2H, +O	vw
TP-R4	108.00	789.4754	789.4764	C_42_H_70_O_12_Na	+O	w
TP-R5	118.56	771.4633	771.4659	C_42_H_68_O_11_Na	−2H	vw
SAL	142.61	773.4815	773.4815	C_42_H_70_O_11_Na		vs
RLM						
TP-H1	44.33	821.4445	821.4453	C_42_H_70_O_13_K	−Na, +2O, +K	vs
TP-H2	53.60	819.4307	819.4296	C_42_H_68_O_13_K	−Na, −2H, +2O, +K	m
TP-H3	61.44	821.4439	821.4453	C_42_H_70_O_13_K	−Na, +2O, +K	vs
TP-H4	65.20	821.4408	821.4453	C_42_H_70_O_13_K	−Na, +2O, +K	s
TP-H5	69.91	819.4313	819.4296	C_42_H_68_O_13_K	−Na, −2H, +2O, +K	w
TP-H6	81.35	805.4508	805.4504	C_42_H_70_O_12_K	−Na, +O, +K	vs
TP-H7	86.37	803.4409	803.4347	C_42_H_68_O_12_K	−Na, −2H, +O, +K	m
TP-H8	90.00	819.4582	819.4507	C_42_H_68_O_14_Na	−2H, +3O	vw
TP-H9	91.57	787.4595	787.4608	C_42_H_68_O_12_Na	−2H, +O	vw
TP-H10	102.10	787.4577	787.4608	C_42_H_68_O_12_Na	−H_2_O, +2O	w
TP-H11	108.00	789.4707	789.4764	C_42_H_70_O_12_Na	+O	vw
SAL	142.61	773.4815	773.4815	C_42_H_70_O_11_Na		w

**Table 4:** Parameters of the ESI-HRMS for the ECR/MS measurements.

Experiments Parameters		Mass Range Parameters	
gas temperature	400 °C	collision energy	40 V
ion source gas 1 (nitrogen)	55 L/min	declustering potential	80 V
ion source gas 2 (nitrogen	55 L/min	mass range	100–800 Da
curtain gas (nitrogen)	45 L/min		
ion spray voltage floating	+5500 V		

**Table 5:** Parameters of the ESI-HRMS for the LC-HRMS measurements.

Experiments Parameters		Mass Range Parameters	
gas temperature	400 °C	MS 1	
ion source gas 1 (nitrogen)	50 L/min	collision energy	10 V
ion source gas 2 (nitrogen)	55 L/min	declustering potential	80 V
curtain gas (nitrogen)	45 L/min	mass range	100–900 Da
ion spray voltage floating	+5500 V	MS 2	
gas temperature	400 °C	collision energy	85 V (LM) 70 V (EC)
		collision energy spread	20 V
		declustering potential	80 V
		mass range	100–900 Da

## Data Availability

Data available on request from the corresponding author.

## Supplementary Materials

The following are available online at https://www.mdpi.com/article/10.3390/antibiotics11020155/s1, Figure S1: Base-peak-chromatogram of EC-treated SAL-solution; Figure S2: Literature known ESI-fragmentation pathway of SAL; Table S1: Optimization of the EC-MS measurements; Table S2: Fragment scheme of fragment ions observed in the MS/MS spectra of SAL; Table S3: Fragment scheme of fragment ions observed in the MS/MS spectra of the EC-TP-5 and EC-TP-7; Table S4: Fragment scheme of fragment ions observed in the MS/MS spectra of the TP-R2 to TP-R4; Table S5: Fragment scheme of fragment ions observed in the MS/MS spectra of the TP-H1 to TP-H6; Table S6: Fragment scheme of fragment ions observed in the MS/MS spectra of the TP-H7 to TP-H11.
