# Supplementary material for: LC-HRMS-Based Identification of Transformation Products of the Drug Salinomycin Generated by Electrochemistry and Liver Microsome
Source: Antibiotics (Basel). 2022 Jan 25;11(2):155. doi: 10.3390/antibiotics11020155 (PMC8868298; doi:10.3390/antibiotics11020155)
Supplement: Supplementary file 1 [file antibiotics-11-00155-s001.zip › antibiotics-1571070-supplementary.pdf]

antibiotics

Supplementary Materials
LC-HRMS based identification of transformation products of the drug Salinomycin generated by electrochemistry and liver microsome

Lisa Knoche [1,2], Jan Lisec [1], Tanja Schwerdtle [2,3] and Matthias Koch [1,*]

1 Department of Analytical Chemistry and Reference Materials, Bundesanstalt für Materialforschung und - prüfung (BAM), Richard-Willstätter-Straße 11, 12489 Berlin, Germany; lisa.knoche@bam.de (L.K.); jan.lisec@bam.de (J.L.)
2 Institute of Nutritional Science, University of Potsdam, Arthur-Scheunert-Allee 114-116, 14558 Nuthetal, Germany; tanja.schwerdtle@uni-potsdam.de
3 German Federal Institute for Risk Assessment (BfR), Max-Dohrn-Str. 8-10, 10589 Berlin, Germany
* Correspondence: matthias.koch@bam.de; Tel.: +49-30-8104-1170

EC/ESI-Single QuadMS – LC-Single QuadMS

Optimization measurements for the electrochemical investigation were performed by ROXYTM system (Antec Scientific, Zoeterwoude, The Netherlands) containing potentiostat and electrochemical flow-through cell. The EC cell consist of a three-electrode arrangement including a titanium auxiliary electrode (inlet-block of the cell), a HyREFTM-reference electrode (Pd/H2) and as working electrode a glassy carbon (GC) or magic diamond (MD). The instrument was controlled via Dialogue software (Antec Leyden) version 2.02.199. The EC-cell was coupled to an electrospray ionization source of a single quadrupole mass spectrometer (Agilent Technologies GmbH, Waldbronn, Germany). Tested parameters were given in Table S1. The LC separation was developed with an Agilent 1290 Infinity system (Agilent Technologies, Waldbronn, Germany), consisting of a 1290 Infinity Sampler, a 1290 Infinity quat. pump, a 1260 Infinity diode array detector, and 1290 Infinity TCC. The system was controlled via OpenLab CDS. The analytical column was a ZorbaxEclipse Plus C18, particle size 1.8 µm, 50x2.1 mm (Agilent Technologies, Waldbronn, Germany).

Table S1. Optimization of the EC-MS measurements, given are tested combinations of electrode, solvents, modifier and potential polarity. (GC: glassy carbon; MD: magic diamond; MeOH: methanol; ACN: acetonitrile; AF: ammonium formiate; FA: formic acid)

electrode	solvent (v/v)	modifier	potential polarity
GC, MD	MeOH:H_2O (2:1)	AF (1 mM), FA (0,1%)	pos
GC, MD	ACN:H_2O (2:1)	AF (1 mM), FA (0,1%)	pos
GC, MD	MeOH:ACN:H_2O (1:3:1) (1:1:1)	AF (1 mM)	pos, neg (only GC)
GC, MD	MeOH:ACN:H_2O (3:1:1)	AF (1 mM. 5mM)	pos, neg (only GC)

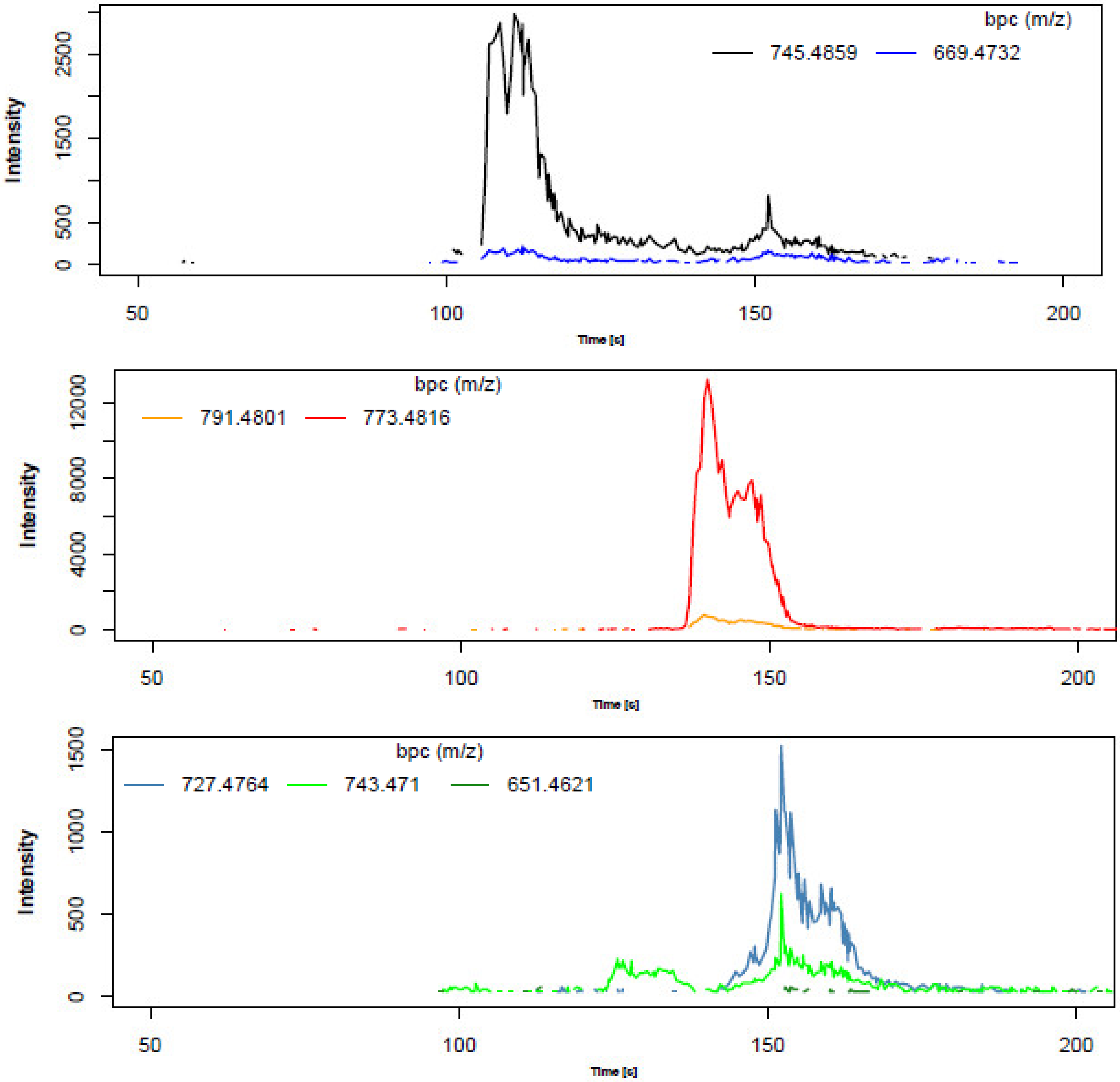

Figure S1. Base-peak-Chromatogram(+ESI, LC-HRMS) of of the EC-treated SAL-solution (GC-electrode). The first chromatogram showed the EC-TP 5 (m/z 745) with co-elution of EC-TP-10 (m/z 669). The second chromatogram showed SAL (m/z 773) with co-elution of EC-TP-1. The third chromatogram showed the EC-TP-7 (m/z 727) with co-elution of EC-TP-6 (m/z 743) and EC-TP-11 (m/z 651).

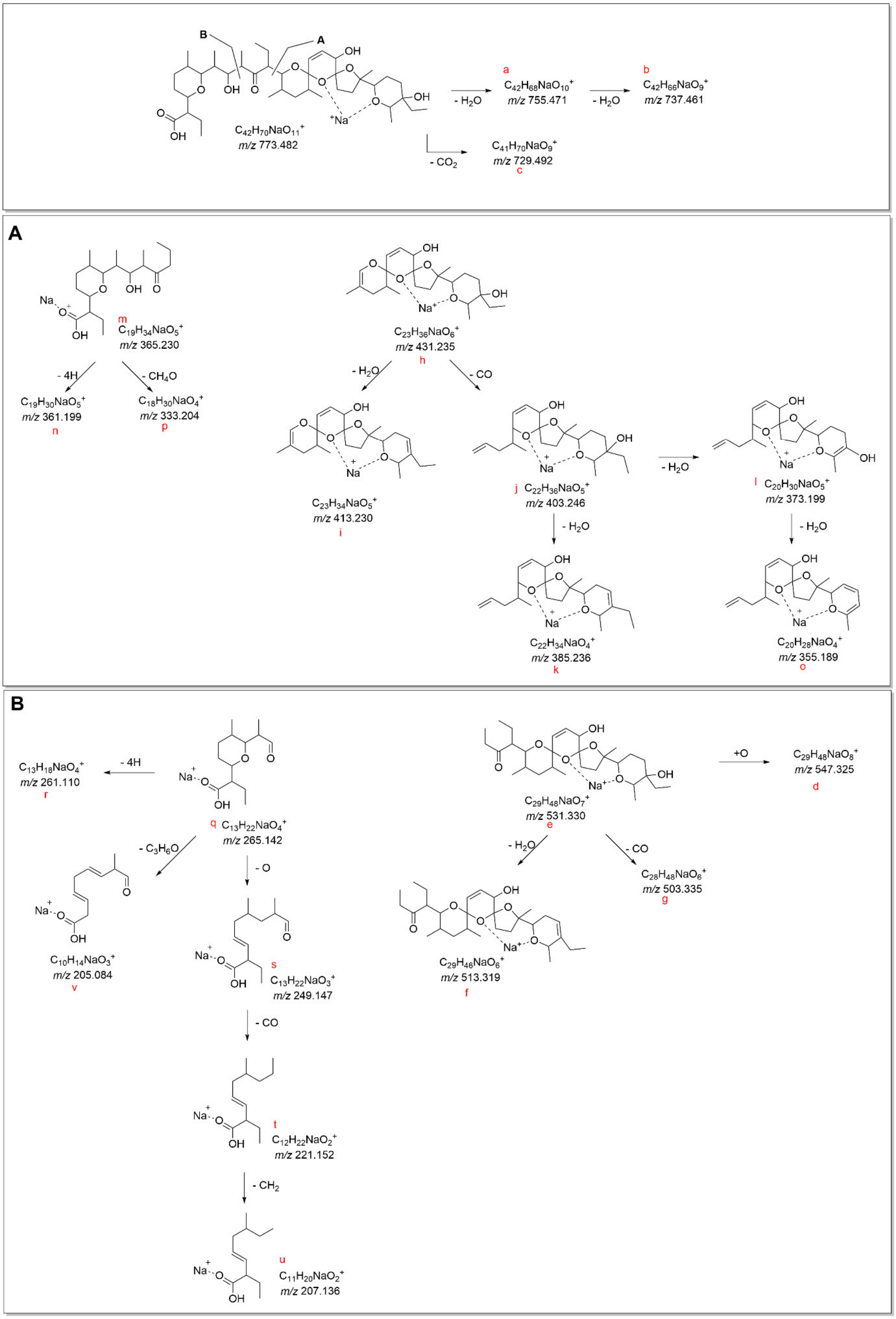

Figure S2. Literature known ESI-fragmentation pathway of SAL, based on the results of Miao et. al. [1]

Table S2. Fragment scheme of fragment ions observed in the MS/MS spectra of SAL (measurement of Std). The intensity (Int) is calculated to the max. intensity of the highest fragment.

	m/z	sf	Int
SAL	773.4816	$C_{42}H_{70}O_{11}Na^+$	26%
a	755.4710	$C_{42}H_{68}O_{10}Na^+$	9%
b	737.4605	$C_{42}H_{66}O_9Na^+$	1%
c	729.4918	$C_{41}H_{70}O_9Na^+$	1%
d	547.3247	$C_{29}H_{48}O_8Na^+$	1%
e	531.3298	$C_{29}H_{48}O_7Na^+$	44%
f	513.3192	$C_{29}H_{46}O_6Na^+$	8%
g	503.3349	$C_{28}H_{48}O_6Na^+$	3%
h	431.2351	$C_{23}H_{36}O_6Na^+$	100%
i	413.2304	$C_{23}H_{34}O_5Na^+$	1%
j	403.246	$C_{22}H_{36}O_5Na^+$	15%
k	385.2355	$C_{22}H_{34}O_4Na^+$	12%
l	373.1991	$C_{20}H_{30}O_5Na^+$	1%
m	365.2304	$C_{19}H_{34}O_5Na^+$	2%
n	361.1991	$C_{19}H_{30}O_5Na^+$	3%
o	355.1885	$C_{20}H_{28}O_4Na^+$	1%
p	333.2042	$C_{18}H_{30}O_4Na^+$	1%
q	265.1416	$C_{13}H_{22}O_4Na^+$	1%
r	261.1103	$C_{13}H_{18}O_4Na^+$	27%
s	249.1467	$C_{13}H_{22}O_3Na^+$	1%
t	221.1517	$C_{12}H_{22}O_2Na^+$	2%
u	207.1361	$C_{11}H_{20}O_2Na^+$	0%
v	205.0841	$C_{10}H_{14}O_3Na^+$	3%

Table S3. Fragment scheme of fragment ions observed in the MS/MS spectra of the EC-TP-5 and EC-TP-7 of the measurements of the glassy carbon generated reaction mix. The intensity (Int) is calculated to the max. intensity of the highest fragment of every TP.

EC-TP-5			EC-TP-7		
#	m/z	int	#	m/z	int
e + CO	531.3356	53%	e + CO	531.3345	51%
e + CO - H_2O	513.3249	3%	e + CO - H_2O	513.3274	3%
e	503.3404	8%	e	503.3414	6%
g	475.3105	1%	f	485.3290	4%
h	431.2462	100%	h	431.2454	100%
i	413.2356	4%	i	413.2360	5%
j	403.2504	31%	j	403.2509	32%
l	373.2050	2%	p	333.2066	1%
n (SAL)	361.2037	2%	q (SAL)	265.1448	20%
m	337.2345	1%	s (SAL)	249.1480	5%
n	333.2078	2%	t - H_2	219.1398	6%
n - C_2H_4	305.1755	1%	u (SAL)	207.1383	8%
n - C_2H_4O	289.1821	2%			
q (SAL)	265.1448	18%			
r (SAL)	249.1503	6%			
q – CO	237.1495	7%			
u (SAL)	207.1386	5%			

v (SAL)	205.0855	1%

Table S4. Fragment scheme of fragment ions observed in the MS/MS spectra of the TP-R2 to TP-R4 of the incubation with rat-liver microsomes. The intensity (Int) is calculated to the max. intensity of the highest fragment of every TP. The intensity of TP-R1 was to low for receiving a MS/MS spectra.

TP-R2			TP-R3		
#	m/z	Int	#	m/z	Int
	805.4719	62%		787.4715	11%
a-805	787.4566	26%	a-787	769.4734	11%
e-805	563.3116	24%	e-SAL-Na	531.3379	33%
e-SAL-K	547.3175	79%	f-SAL-Na	513.3321	12%
e-SAL-Na	531.3481	9%	h-SAL-Na	431.2500	100%
f-SAL-K	529.3021	17%	i-SAL-Na	413.2373	30%
h-805	463.2242	9%	j-SAL-Na	403.2540	48%
h-SAL-K	447.2268	100%	q-787	279.1242	20%
h-SAL-Na	431.2488	38%	q-SAL-Na	265.1413	17%
i-SAL-K	429.2143	9%	s-SAL-Na	249.1475	11%
i-SAL-Na	413.2422	12%	u-SAL-Na	207.1384	10%
j-SAL-Na	403.2527	22%			
m-805	397.1961	15%			
n-SAL-K	377.1826	12%			
q-789	281.1416	9%			
q-SAL-K	281.1161	6%			
TP-R4			TP-R5		
#	m/z	Int	#	m/z	Int
	789.501	6%		771.4858	9%
a-789	771.4887	4%	a-771	753.4757	2%
e-789	547.3395	28%	e-SAL-Na	531.3427	32%
e-SAL-Na	531.3363	2%	f-SAL-Na	513.3332	12%
f-789	529.3261	6%	g-SAL-Na	503.3484	7%
g-789	519.3415	2%	h-SAL-Na	431.2505	100%
h-789	447.2466	100%	i-SAL-Na	413.2375	26%
i-789	429.2328	18%	j-SAL-Na	403.2539	39%
j-789	419.2507	20%	l-SAL-Na	373.2058	7%
l-789	389.1955	2%	p-SAL-Na	333.2052	5%
n-789	377.2022	2%	q-SAL-Na	265.1452	11%
m-SAL-Na	365.236	4%	q-771	263.1304	21%
n-SAL-Na	361.2093	2%	s-SAL-Na	249.1553	5%
q-789	281.1408	8%	u-SAL-Na	207.1391	7%
s-789	265.1455	36%			
u-789	223.132	3%			
v-789	221.1546	2%			

Table S5. Fragment scheme of fragment ions observed in the MS/MS spectra of the TP-H1 to TP-H6 of the incubation with human-liver microsomes. The intensity (Int) is calculated to the max. intensity of the highest fragment of every TP.

TP-H1			TP-H2		
#	m/z	Int	#	m/z	Int
	821.4661	80%	e-805	819.4616	78%
a-821	803.4577	23%	e-SAL-K	563.3056	90%
b-821	785.4391	11%	h-805	547.3378	66%
e-821/d-805	579.3065	15%	h-SAL-K	463.2145	61%
e-805	563.3097	100%	l-SAL-K	447.2431	52%
f-805	545.2937	9%	n-SAL-K	389.1993	9%
h-805	463.2183	50%	n-SAL-Na	377.1749	44%
i-805	445.2112	7%	q-803	361.2043	100%
m-805	397.2021	7%	e-805	295.1575	36%
n-SAL-K	377.1784	51%			
n-SAL-Na	361.2079	9%			
n-SAL-K-H_2O	359.1635	7%			
q-805	297.1056	6%			
q-789Na	281.1388	8%			
q-SAL-K	281.1166	9%			
**TP-H3**			**TP-H4**		
#	m/z	Int	#	m/z	Int
	821.4629	52%	a-821	821.4686	70%
Na-H2	803.467	24%	e821/d-805	803.4512	62%
a-821	803.4388	18%	e-805	579.3111	15%
e-805	563.3080	48%	f-805	563.3052	100%
f-805	545.2965	18%	h-805	467.2525	17%
h-805	463.2192	100%	i-805	463.2238	83%
h-Sal-K	447.2476	11%	q-805	445.2049	6%
i-805	445.207	26%	a-821	297.1059	19%
i-SAL-K	429.2365	7%			
j-SAL-K	419.2439	5%			
q-805	297.12	5%			
s-805	281.1399	11%			
**TP-H5**			**TP-H6**		
#	m/z	Int	#	m/z	Int
	819.4456	73%		805.473	50%
e-805	563.3084	89%	a-805	787.4576	21%
e-803	561.2938	24%	e-805	563.3133	12%
e-SAL-K	547.3303	46%	e-SAL-K	547.3128	64%
f-805	545.3053	11%	f-SAL-K	529.3027	27%
h-805	463.2163	76%	h-SAL-K	447.2208	100%
h-SAL-K	447.2415	100%	i-789-Na	429.2316	11%
i-803	443.2453	22%	i-SAL-K	429.2125	15%
j-SAL-K	419.2473	37%	j-SAL-Na	403.252	14%
q-SAL-K	281.141	39%	m-805	397.205	5%
			q-SAL-K	281.1403	8%

Table S6. Fragment scheme of fragment ions observed in the MS/MS spectra of the TP-H7 to TP-H11 of the incubation with human-liver microsomes. The intensity (Int) is calculated to the max. intensity of the highest fragment of every TP.

TP-H7			TP-H8		
#	m/z	Int	#	m/z	Int
	803.4604	45%		819.4557	100%
a-803	785.4384	32%	531 + 3 O -H_2O	561.2818	70%
e-SAL-K	547.3132	57%	e-789	547.3144	12%
e-SAL-Na	531.3417	38%	h-789	447.2294	43%
e-SAL-Na -		12%			
2H	529.325		h-SAL-K	447.2018	29%
h-SAL-K	447.2216	66%			
h-SAL-Na	431.2534	100%			
i-SAL-K	429.2248	6%			
i-SAL-Na	413.2393	29%			
j-SAL-Na	403.2479	22%			
s-789	265.1496	14%			
**TP-H9**			**TP-H10**		
#	m/z	Int	#	m/z	Int
	787.4769	8%		787.4824	17%
a-787	769.4609	8%	d-787	561.3107	11%
e-SAL-Na	531.3408	34%	f-789	529.3223	100%
f-SAL-Na	513.3308	17%	f-789 -CO_2	485.3291	11%
g-SAL-Na	503.3387	10%	i-789	429.2341	10%
h-SAL-Na	431.2479	100%	n-SAL-Na -O	345.2121	4%
i-SAL-Na	413.2376	30%	q789	281.1438	14%
j-SAL-Na	403.2507	27%	s-789	265.1438	25%
m-787+2H	379.2195	4%			
p-SAL-Na	333.2031	5%			
q-787	279.1255	14%			
q-SAL-Na	265.1377	8%			
u-SAL-Na	207.1381	8%			
**TP-H11**					
#	m/z	Int			
	789.485	22%			
e-789	547.3348	45%			
e-SAL-Na	531.3334	9%			
f-789	529.3314	14%			
f-SAL-K	529.312	9%			
h-789	447.2438	100%			
i-789	429.2314	25%			
j-489	419.2477	47%			
q-789	281.1447	15%			
s-789	265.1434	55%			

References

1. Miao, X.S.; March, R.E.; Metcalfe, C.D. Fragmentation study of salinomycin and monensin A antibiotics using electrospray quadrupole time-of-flight mass spectrometry. *Rapid Commun Mass Spectrom* **2003**, *17*, 149-154, doi:10.1002/rcm.882.
